# Mitochondrial DNA-Mediated Inflammation in Acute Kidney Injury and Chronic Kidney Disease

**DOI:** 10.1155/2021/9985603

**Published:** 2021-06-29

**Authors:** Lini Jin, Binfeng Yu, Ines Armando, Fei Han

**Affiliations:** ^1^Kidney Disease Center, The First Affiliated Hospital, Zhejiang University School of Medicine, Institute of Nephrology, Zhejiang University, Key Laboratory of Kidney Disease Prevention and Control Technology, Hangzhou, Zhejiang, China; ^2^Department of Medicine, School of Medicine and Health Sciences, The George Washington University, Washington, D.C., USA

## Abstract

The integrity and function of mitochondria are essential for normal kidney physiology. Mitochondrial DNA (mtDNA) has been widely a concern in recent years because its abnormalities may result in disruption of aerobic respiration, cellular dysfunction, and even cell death. Particularly, aberrant mtDNA copy number (mtDNA-CN) is associated with the development of acute kidney injury and chronic kidney disease, and urinary mtDNA-CN shows the potential to be a promising indicator for clinical diagnosis and evaluation of kidney function. Several lines of evidence suggest that mtDNA may also trigger innate immunity, leading to kidney inflammation and fibrosis. In mechanism, mtDNA can be released into the cytoplasm under cell stress and recognized by multiple DNA-sensing mechanisms, including Toll-like receptor 9 (TLR9), cytosolic cGAS-stimulator of interferon genes (STING) signaling, and inflammasome activation, which then mediate downstream inflammatory cascades. In this review, we summarize the characteristics of these mtDNA-sensing pathways mediating inflammatory responses and their role in the pathogenesis of acute kidney injury, nondiabetic chronic kidney disease, and diabetic kidney disease. In addition, we highlight targeting of mtDNA-mediated inflammatory pathways as a novel therapeutic target for these kidney diseases.

## 1. Introduction

Mitochondria are double membrane-bound organelles that appear in nearly all eukaryotic cells. In addition to adenosine triphosphate (ATP) production, mitochondria participate in multiple physiological processes, such as heat production, redox homeostasis, calcium signaling, cell growth and death pathway, and antimicrobial immunity [1, 2]. Considering its essential role in providing energy, the integrity and normal function of mitochondria are crucial for the normal function of cells, especially in organs that need a lot of energy, such as the heart and kidney. When the mitochondria are injured, a variety of mitochondrial components will be released into the cytoplasm or extracellular environment and recognized as damage-associated molecular patterns (DAMPs) by pattern recognition receptors (PRRs), thus promoting downstream proinflammatory responses [3, 4]. Although many other mitochondrial components such as *N*-formyl peptides, ATP, and cardiolipin can act as mitochondrial DAMPs, we focus on mitochondrial DNA (mtDNA) in this review.

mtDNA derives from ancestral bacterial genome and has a double-strand circular structure, 16.5 kb in length. The copy number of mtDNA varies among different cell types, ranging from 100 to 10000 [5]. Mammalian mtDNA encodes 11 messenger RNAs which can be translated to 13 proteins forming four oxidative phosphorylation (OXPHOS) complexes [6]. Although a delicate quality control system has evolved to maintain mitochondrial homeostasis [2], mtDNA is particularly vulnerable to oxidative damage compared with nuclear DNA, due to its subcellular localization close to the electron transport chain where ROS is generated and the lack of protective histones. Mitochondrial genome damage or mutation may lead to aerobic respiration disruption, cellular dysfunction, and even cell death. Accumulating evidence suggests that mtDNA may contribute to the activation of innate immune response which acts as the central hub of the pathogenesis of many diseases [7].

Acute kidney injury (AKI) is characterized by a rapid decline of kidney function, which may progress to chronic kidney disease (CKD) and end-stage renal disease (ESRD). It remains a global challenge for its high morbidity and mortality [8, 9]. The main causes of AKI include renal ischemia, sepsis, and nephrotoxicity. The pathogenesis of AKI and CKD is still unclear, although a key role of prolonged or excessive inflammation has been recognized for a long time. In mechanism, tubular epithelial cell damage has been found pivotal to initiate the inflammatory response via activating resident immune cells such as macrophages and infiltrating leukocytes in the kidney, which release inflammatory mediators to amplify the cascades [10–13]. Moreover, recent studies have demonstrated that mtDNA-associated inflammatory responses were implicated in the pathogenesis of AKI and progression of CKD [14–16].

In this review, we summarize the current understanding of how abnormal mtDNA drives innate immunity and of its role in renal inflammation and in the development of several kidney diseases, including AKI, nondiabetic CKD, and diabetic kidney disease (DKD). In addition, we highlighted the potential of mtDNA as a new indicator as well as a putative therapeutic target for these disorders.

## 2. mtDNA-Sensing Mechanisms

**Figure 1:**
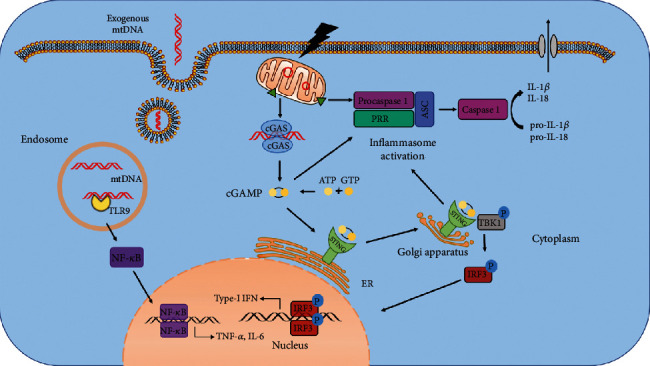
Overview of mtDNA-sensing pathways mediating inflammatory cascades. In conditions of cell injury or stress, aberrant mtDNA is released out of the mitochondria and is recognized by three major sensors to drive innate immune responses. Firstly, TLR9 binds to and is activated by mtDNA in the endosome facilitating downstream NF-*κ*B, leading to upregulated expression of proinflammatory cytokines such as TNF-*α* and IL-6. Cytosolic mtDNA is also recognized by cGAS which results in the translocation of STING from the ER to the Golgi apparatus, resulting in TBK1-IRF3 activation and increased type-I IFN expression. In addition, mislocalized mtDNA also activates PRRs like NLRP3, which recruits ASC and procaspase 1 to form the inflammasome and contributes to cleavage of IL-1*β* and IL-18 to their mature forms. ASC, adaptor protein of apoptosis-associated speck-like protein containing a caspase recruitment domain; ATP, adenosine triphosphate; cGAMP, cyclic guanosine monophosphate-adenosine monophosphate; cGAS, cyclic GAS; ER, endoplasmic reticulum; GTP, guanosine triphosphate; IFN, interferon; IRF3, IFN regulatory factor 3; mtDNA, mitochondrial DNA; PRR, pattern recognition receptor; STING, stimulator of interferon genes; TBK1, TANK-binding kinase 1; TLR9, Toll-like receptor 9.

Although mtDNA is an inherent component of the mitochondria, it can be recognized in the cytosol to trigger innate immunity by different mechanisms because it is relatively isolated, as shown in Figure 1. Moreover, growing evidence also suggests that underlying interactions exist between these DNA-sensing pathways.

### 2.1. mtDNA and Toll-Like Receptor 9 (TLR9)

Toll-like receptors (TLRs) belong to highly conserved PRRs that play essential roles in recognizing pathogen-associated molecular patterns (PAMPs) and in triggering innate immune responses and inflammatory cascades [17–19]. Among them, TLR9 has been shown pivotal in sensing bacterial DNA, particularly, unmethylated cytosin-guanosin dinucleotide (CpG) DNA, to provoke innate immunity [20, 21]. Mechanistically, the binding of TLR9 to bacterial CpG DNA is preceded by the endocytosis of foreign bacteria and subsequent translocation of TLR9 from the endoplasmic reticulum to the endocytic vesicles [19, 22, 23]. Evolutionarily derived from bacterial DNA, mtDNA thereby retains unmethylated CpG motifs as well as the ability to activate the TLR9 pathway in a parallel way [24].

The interaction between mtDNA and TLR9 has been shown to be involved in the development of a variety of disorders, such as acute myocardial infarction [25], hepatocellular carcinoma [26, 27], nonalcoholic steatohepatitis [28, 29], and sterile lung injury [30, 31]. Typically, mislocated mtDNA activates TLR9 signaling and downstream myeloid differentiation factor 88 (MyD88), resulting in upregulated expression of nuclear factor- (NF-) *κ*B and other proinflammatory factors such as tumor necrosis factor-*α* and interleukin 6, to amplify inflammation and exaggerate cell damage [31]. Besides, circulating mtDNA was reported to stimulate TLR9 in polymorphonuclear neutrophils, then facilitate p38-MAPK pathway and contribute to neutrophil secretion [4].

### 2.2. mtDNA and cGAS-STING Signaling Pathway

Over the past few years, cyclic guanosine monophosphate- (GMP-) adenosine monophosphate (AMP) (cGAMP) synthase (cGAS) has been identified as an important cytosolic DNA sensor which can elicit type-I interferon (IFN) signaling in mammalian cells. Under the condition of cell stress or cell injury, self-DNA from the nucleus or the mitochondria may leak into the cytosol and sensitize cGAS, which further converts ATP and GTP to cyclic GAMP, a second messenger mediating the activation of stimulator of interferon genes (STING). Thereafter, stimulated STING traffics from the endoplasmic reticulum membrane to the Golgi apparatus and interacts with I*κ*B kinase- (IKK-) related kinase TANK-binding kinase 1 (TBK1), which phosphorylates IFN regulatory factor 3 (IRF3) to induce type-I IFN expression [32–35]. The cGAS-STING signaling pathway has been extensively recognized as the predominant pathway of DNA sensing and immune defense in a number of infectious diseases caused by various pathogens. Besides, cGAS acts as the first line of antitumor defense since it can sense the cytosolic DNA of antigen-presenting cells or tumor cells and trigger an antitumor immune response. Cellular senescence, autoimmune diseases, and heart failure are also associated with self-DNA-mediated cGAS-STING activation [36, 37].

Cytosolic mtDNA is one of the main causes for the activation of the cGAS-STING pathway. To date, it has been widely accepted that Bak/Bax-dependent mitochondrial outer membrane permeabilization (MOMP) initiates the release of mtDNA and thus contributes to cGAS-STING-mediated DNA-sensing pathway. In 2018, an Australian group using live-cell lattice light-sheet microscopy found that Bak/Bax pores forming on the outer membrane led to extrusion of the inner mitochondrial membrane into the cytosol, which carries the mitochondrial genome [38]. Later, a group in UK using superresolution imaging showed that during cell death, mitochondrial inner membrane permeabilization (MIMP) occurred following MOMP and allowed mtDNA efflux [39]. On the other hand, a recent study showed that in nonapoptotic cells, small mtDNA fragments were released through pores in the mitochondrial outer membrane (MOM) formed by voltage-dependent anion channel (VDAC) oligomers. Under moderate oxidative stress, the negatively charged backbone of mtDNA interacts directly with positively charged N-terminal domain of VDAC1 to facilitate VDAC1 oligomerization and increase mtDNA release, which drives the IFN signaling response and contributes to the pathogenesis of autoimmune diseases [40, 41]. Additionally, Tigano et al. described that mtDNA double-strand breaks (mtDSBs) triggered type-I IFN response through a novel intrinsic immune surveillance mechanism by which herniation formed by Bak and Bax released mitochondrial RNA into the cytoplasm and activated RIG-I–MAVS signaling pathway [42].

The mitochondrial transcription factor A (TFAM) is an essential protein required for the transcription and replication of mtDNA. Normally, TFAM, along with mtDNA and other proteins, constitutes nucleoid and regulates its architecture, abundance, and segregation. West et al. revealed that TFAM deficiency could promote mitochondrial stress and lead to abnormal mtDNA packaging, which would be released into the cytosol and then trigger cGAS-STING to elicit antiviral signaling [43].

### 2.3. mtDNA and Inflammasomes

Inflammasomes are multiprotein complexes and are well known for their fundamental roles in caspase activity, innate immunity, and cell death. Canonical inflammasomes consist of PRRs, the adaptor protein of apoptosis-associated speck-like protein containing a caspase recruitment domain (CARD) (ASC) and procaspase 1. Upon activation by PAMPs or DAMPs, PRRs assemble and activate caspase 1, which promotes the maturation of proinflammatory cytokines IL-18 and IL-1*β*, as well as the cleavage of gasdermin D (GSDMD), leading to pyroptosis, an inflammatory form of regulated necrosis [44, 45]. Nucleotide-binding oligomerization domain- (NOD-) like receptor (NLR) and absent in melanoma 2- (AIM2-) like receptors (ALR) are two out of five PRRs that form inflammasomes. Particularly, NLRP3 inflammasome and AIM2 inflammasome have been frequently linked to mtDNA signaling.

NLRP3 inflammasome constitutes a substantial part of innate immune defense against various infections and participates in the pathophysiology of multiple inflammatory diseases [46, 47]. Leakage of mtDNA [48, 49], as well as other stimuli such as K+ efflux [50] and mitochondrial ROS production [51], is sufficient to trigger the NLRP3 inflammasome cascades. Specifically, cytosolic mtDNA binds to and activates NLRP3 inflammasome in an oxidized form [48]. Further evidence also suggests that mtDNA is released out of mitochondria in a NLRP3 inflammasome-dependent way [52]. Therefore, a positive feedback between mtDNA release and NLRP3 inflammasome activation may reinforce the inflammatory process and enhance tissue damage.

AIM2 senses double-strand DNA (dsDNA), rather than single-strand DNA or RNA, and elicits inflammasome assembly and activation. Endogenous dsDNA derived from irradiation or chemotherapy-induced DNA damage has been implicated in cell death mediated by AIM2 inflammasome [53–56]. In addition to intracellular cytosolic “self-DNA,” exosome secretory and circulatory cell-free mtDNA are also suggested to contribute to AIM2 inflammasome-mediated inflammation [57].

### 2.4. The Interplay among Different mtDNA-Sensing Pathways

The cGAS-STING pathway and inflammasome activation have been shown to be associated in multiple sets, such as acute lung injury [58] and age-related liver ischemia-reperfusion injury (IRI) [59]. Typically, the stimulated cGAS-STING axis initiates inflammasome assembly and activation via type-I IFN signaling [60, 61] or K+ efflux induced by the translocation of STING to the lysosome and the subsequent lysosomal rupture [62]. In LPS-induced cardiomyopathy, STING-phosphorylated IRF3 traffics to the nucleus and increases the expression of NLRP3, providing the priming signal of inflammasome activation [63]. However, activation of inflammasome has been suggested to suppress the cGAS-STING pathway [64]. Wang et al. found that in response to DNA virus infection, canonical or noncanonical inflammasome activation led to caspase-1 or caspase-4, caspase-5, and caspase-11-dependent cleavage of cGAS and reduced IFN production [65]. Moreover, Banerjee et al. showed that AIM2 inflammasome-activated GSDMD formed pores on the cell membrane and induced K+ efflux, causing a decrease of intracellular K+ which undermined the DNA binding capacity and enzymatic activity of cGAS [66]. Notwithstanding, IL-1*β*, the product of inflammasome activation and pyroptosis, was found to induce mtDNA release and activate cGAS-STING signaling which protected cells against RNA virus infection [67]. Therefore, the complex positive and negative relationships between the cGAS-STING pathway and inflammasome activation remain elusive and need further investigation.

TLR9-mediated NLRP3 inflammasome activation has been described in several disease models [68–70]. However, the mechanisms of this interrelationship have not been fully elucidated. Besides, the DNA-sensing cGAS-STING and TLR9 signaling pathways were suggested in limited studies to work synergistically in innate immune response [71, 72].

## 3. mtDNA and Kidney Diseases

### 3.1. mtDNA and AKI

The significance of the integrity and function of mitochondria for normal kidney function has been universally established. As a key indicator of mitochondrial function, mtDNA copy number (mtDNA-CN) abnormalities have been frequently observed during the development of AKI in both animal models and clinical trials, as shown in Table 1. In the murine model of LPS-induced kidney injury, the mtDNA-CN of whole cell lysates declined [73], while the mtDNA-CN of cytoplasmic extracts increased [15], probably indicating that under cell stress, mtDNA replication was restricted but preexisting mtDNA continued to be released from the mitochondria to the cytosol. Analysis of circulating mtDNA-CN revealed that the concentration of mtDNA in plasma tended to increase although not significantly in bilateral ureteral obstruction (BUO) and ischemia-reperfusion models in mice [16]. Compared with circulating mtDNA-CN, urinary mtDNA- (UmtDNA-) CN has greater potential as an ideal indicator for AKI owing to its accessibility, correlation with renal function, and predictive value for kidney prognosis [74–76]. A case-control study on systemic inflammatory response syndrome (SIRS) showed that increased circulating mtDNA was not related to the renal function, whereas the level of UmtDNA correlated with the severity of AKI. The study also demonstrated that tubular epithelial cells expressed proinflammatory cytokines in response to mtDNA intervention [77].

Several studies have been conducted to assess whether and how aberrant mtDNA contributes to renal inflammation and the onset of AKI. Early in 2008, Yasuda et al. showed that TLR9 deficiency or TLR9 suppression by a selective inhibitor H154 protected mice from septic AKI as evidenced by increased survival, improved kidney function, and decreased inflammatory cytokine release and splenic apoptosis [78]. In 2016, the same group found that mice intravenously injected with exogenous mitochondrial debris presented with kidney injury, mitochondrial damage, and cytokine production, which were reversed in *Tlr9* KO mice or by pretreatment with DNase [14]. Their results suggested that mtDNA facilitated TLR9 activation and contributed to septic AKI. However, global *Tlr9* deletion in mice had no protective effect on ischemic kidney injury [79, 80], while renal proximal tubule specific deficiency or inhibition of TLR9 significantly ameliorated renal damage and dysfunction after renal ischemia [81, 82]. The different outcomes imply diverse functions of TLR9 depending on the specific cell types which merits further investigation. In cisplatin-induced AKI, the expression of cGAS and STING is enhanced, accompanied by increased phosphorylation of downstream TBK1 and p65, and translocation of STING to the Golgi apparatus. Depletion of STING utilizing knockout mice and pharmacological inhibition of STING by C-176 both alleviated inflammatory responses and improved renal dysfunction. However, the classic downstream molecules including IRF3 and type-I IFNs remained unchanged, which needed to be further clarified [15]. In addition, mitochondrial damage and NLRP3 inflammasome activation were described in AKI models caused by contrast media. Inhibition of PINK1-parkin pathway-mediated mitophagy enhanced the generation of mt-ROS and NLRP3 inflammasome activation in human renal proximal tubular cell line (HK2 cell), which could be attenuated by the administration of MitoTEMPO, a mitochondria-targeted antioxidant. However, only oxidized nuclear DNA but not mtDNA was analyzed in this experimental setting [83].

### 3.2. mtDNA and Nondiabetic CKD

Increasing evidence suggests that mtDNA-CN is closely correlated with the progression of CKD (Table 1). Decreased mtDNA content was observed in the renal cortex of partially nephrectomized rats, a commonly used CKD model [84]. The Atherosclerosis Risk in Communities (ARIC) study showed that higher mtDNA-CN level in the buffy coat was associated with decreased incidence of CKD independent of traditional risk factors such as diabetes and hypertension [85]. In agreement, a recent cohort study involving 4812 CKD patients demonstrated that decreased mtDNA-CN in whole blood correlated with increased all-cause mortality and infection-related deaths [86]. The level of mtDNA-CN in blood cells is negatively correlated with the occurrence and prognosis of CKD, whereas cell-free circulating mtDNA-CN tends to be positively correlated to kidney injury [16]. Of note, cell-free circulating mtDNA was also detected in abundance in healthy individuals [87]. Basically, the role of cell-free circulating mtDNA is not well understood, since the quality other than the quantity of mtDNA is rarely evaluated, and damages to mtDNA such as oxidation, fragmentation, and break may be more direct triggers to act as DAMPs. Moreover, elevated UmtDNA was detected in patients with hypertension compared with that in healthy individuals, and this elevation was associated to markers of kidney damage [88]. In a longitudinal study, analysis of 131 CKD patients showed that lower UmtDNA at baseline was linked to favorable renal outcomes at 6 months [89]. Similarly, an observational study involving 32 nondiabetic CKD patients showed that the level of UmtDNA correlated with the rate of renal function decline and predicted the risk of serum creatinine doubling or need of dialysis [90]. However, in a larger cohort of 102 nondiabetic CKD patients, there was no significant correlation between the UmtDNA level and the rate of eGFR decline, though the UmtDNA level was associated with baseline eGFR, proteinuria, and pathological damage [91]. Based on these results, whether UmtDNA can serve as a reliable indicator of CKD progression remains to be determined.

Abnormalities in mtDNA may also enable renal inflammation and fibrosis and promote CKD progression. TFAM-associated mitochondrial dysfunction is involved in the development of various kidney diseases including cisplatin-induced AKI [92], CKD [93], and kidney cystic disease [94]. Chung et al. demonstrated that mice with conditional knockout of *Tfam* in kidney tubule cells presented with mtDNA depletion and bioenergetic impairment at 6 weeks and renal fibrosis, immune cells infiltration, and azotemia at 12 weeks. Mechanistically, TFAM deficiency causes mtDNA mispackaging and leaking into the cytosol resulting in the activation of cGAS-STING pathway and the upregulation of downstream NF-*κ*B which underlies the renal fibrosis and inflammation in CKD progression [93].

Mitochondrial dysfunction and the subsequent NLRP3 inflammasome activation have been linked to renal tubular injury and tubulointerstitial fibrosis in albumin-overload mouse models and aldosterone-treated human tubular epithelial cells [95, 96]. In the CKD models of nephrectomy and unilateral ureteral obstruction (UUO), *Nlrp3* knockout ameliorated mitochondrial morphological abnormalities and mtDNA-CN reduction, thus attenuating renal fibrosis [97, 98]. Similarly, administration of cyclosporin A (CsA), a mitochondrial permeability transition pore (mPTP) inhibitor, also attenuated mitochondrial damage and NLRP3 inflammasome activation [95]. Early studies demonstrated that mtDNA fragments could release into the cytosol through mPTP, and CsA prevented pore opening and subsequent mtDNA release [99, 100]. Taken together, these findings suggest that cytosolic mtDNA contributes to CKD progression via activation of NLRP3 inflammasome.

### 3.3. mtDNA and Diabetic Kidney Disease (DKD)

DKD is the leading cause of CKD and ESRD worldwide. DKD patients are predisposed to cardiovascular diseases, infections, and mortality [101]. Nevertheless, the pathogenesis of DKD is still elusive. Complications of diabetes in organs other than the kidney, such as diabetic retinopathy [102], diabetic peripheral neuropathy [103], and skin conditions [104] have been associated with mitochondrial dysfunction and mtDNA changes. The involvement of oxidative stress and mtDNA damage is also gradually recognized as a key factor underlying the development of DKD. About 20 years ago, hyperglycemia-induced oxidative mtDNA damage was found implicated in diabetic nephropathy (DN), as evidenced by increased 8-OHdG expression and subsequent mtDNA deletion [105, 106]. Using gas chromatography-mass spectrometry, Sharma et al. found that most of the differentially expressed urine metabolites in DKD patients in comparison with those in healthy individuals were linked to mitochondrial functions. Reduced mtDNA in urine exosomes, which reflected intrarenal mtDNA levels, further confirmed mitochondrial damage in DKD [107]. Moreover, the mtDNA levels in urine supernatants negatively correlated with intrarenal mtDNA levels and might serve as a potential indicator of the severity of interstitial fibrosis in patients pathologically diagnosed with DN [108]. However, the alteration of peripheral blood mtDNA in DKD patients is controversial. An early study found increased peripheral blood mtDNA-CN in type-2 diabetic patients with nephropathy, compared with that in those patients without nephropathy and in healthy controls [109], whereas another result recently showed a low peripheral blood mtDNA-CN in DN patients [110]. Therefore, large-scale, long-term studies are still needed to determine the significance of mtDNA changes in peripheral blood in DKD patients.

High glucose downregulated intracellular mtDNA in murine endothelial cells and podocytes and facilitated the release of mtDNA into circulation which filtered through the kidney and further triggered chronic renal inflammation [111]. However, human mesangial cells treated by high glucose showed elevated cellular mtDNA content, accumulation of ROS, and increased mitochondrial fragmentation [112]. After that, excessive ROS caused mtDNA damage and activated TLR9 pathway as evidenced by increased expression of NF-*κ*B and MYD88 [113]. It was also suggested that altered mtDNA content and mtDNA damage occurred earlier than bioenergetic dysfunction. These results indicate that mtDNA change in mesangial cells may contribute to the development of DKD.

## 4. Therapeutic Targets and Future Perspectives

A series of intracellular mechanisms, including mt-ROS scavenging, mitochondrial biogenesis, mitophagy, and mtDNA repair, are among the mitochondrial quality control system, which work synergistically to maintain mitochondrial homeostasis [2]. Since the common priming step of mtDNA-sensing pathways mediating inflammatory responses is mtDNA damage or release, it can be theorized that protective strategies specific to mtDNA or mitochondria may be preferred choices for the treatment of kidney injury, as shown in Table 2. Firstly, multiple mitochondria-targeted antioxidants, such as mitoquinol mesylate (MitoQ), SS-31, or plastoquinol decylrhodamine 19 (SkQR1), have been shown to effectively attenuate ROS accumulation and kidney injury and favor recovery of kidney function [114–117]. In IR-induced AKI, administration of MitoTEMPO, a specific scavenger of mitochondrial superoxide, alleviated mitochondrial damage and inflammation, partially by rescuing TFAM transcription decrease and mtDNA depletion caused by excess mt-ROS [118]. Additionally, treatment with diazoxide, a mitochondrial K_ATP_ channel opener, was also found to reduce ROS accumulation and mtDNA oxidization and thereby ameliorate renal ischemic injury [119].

Mitochondria are constantly renewed through eliminating the old or damaged ones by mitophagy and producing new, functional mitochondria by mitochondrial biogenesis. Peroxisome proliferator-activated receptor gamma coactivator (PGC-1*α*) is the master regulator of mitochondrial biogenesis and is highly expressed in the kidney, making it a potential therapeutic target for different kidney diseases [120, 121]. Treatment with formoterol, a specific *β*_2_-adrenergic agonist, stimulated mitochondrial biogenesis and facilitated the recovery of renal function via upregulation of PGC1-*α* following IR-induced AKI in mice [122]. In addition, agonism of 5-hydroxytryptamine 1F (5-HT_1F_) receptors also increased PGC1-*α* transcript levels and restored the expression of mitochondrial proteins and mtDNA-CN altered by AKI [123, 124].

However, there is still a lack of treatments directly targeting mtDNA [125, 126]. In spite of limited self-repair capacity of mtDNA compared with that of nucleus DNA, several mtDNA repair mechanisms have already been determined, including base excision repair, DNA break repair, mismatch repair, and homologous recombination (HR) [127, 128]. In the kidney, some key molecules involved in mtDNA maintenance have also been identified, such as polymerase *β* and TWNK [129, 130]. In addition, 8-hydroxyguanine DNA glycosylase (OGG1), an mtDNA repair protein, was elevated in the kidneys of mice with septic AKI [131]. Besides, Y-box-binding protein 1 (YBX1), which mediated mismatch mtDNA repair, was upregulated in the kidneys of CKD and DKD patients and UUO mouse models [132]. Moreover, a recent study on rheumatoid arthritis revealed that inhibition of MRE11A, a DNA repair nuclease located in mitochondria, resulted in mtDNA oxidization and translocation, triggering inflammasome assembly and tissue inflammation, which were reversed by MRE11A overexpression [133]. However, whether interventions on the mtDNA repair proteins would make a difference in the progression of AKI and CKD remains to be investigated. In addition, suppressing the release of mtDNA appears to be another option to restrain the downstream inflammation, taking into account the already unveiled mechanisms, including Bak/Bax-dependent MOMP, VDAC oligomers-formed pores, and mPTP.

The detection technology of mtDNA-CN is another noteworthy issue. Until now, the most widely used method to measure mtDNA-CN is quantitative polymerase chain reaction (qPCR), by calculating the ratio of copy number of a mitochondrial gene to that of a nuclear gene [134]. The limitation of qPCR for mtDNA-CN measurement is that it can only quantify a relative copy number. Recently, digital PCR (dPCR) has emerged as a new method to quantify absolute mtDNA-CN [90, 91]. Other methods like plasmid-normalized PCR-based assay and DNA microarrays have also been tried to estimate mtDNA-CN [85, 86]. Therefore, more technological advances are needed in the future for high accuracy and convenience of mtDNA-CN measurement.

In conclusion, mitochondrial dysfunction and mtDNA abnormality are associated with AKI and CKD, and mtDNA-CN may be a potential biomarker for the assessment of kidney injury and for the prediction of renal prognosis. Moreover, mtDNA leaking into the cytoplasm may trigger innate immunity via several DNA-sensing mechanisms including TLR9, cGAS-STING, and NLRP3/AIM2 inflammasome signaling as well as the interactions among these pathways, leading to kidney inflammation and fibrosis which are implicated in the pathogenesis of AKI, nondiabetic CKD, and DKD. Further, strategies targeting mtDNA-sensing pathway-mediated inflammation, including using mitochondria-targeted antioxidants, STING, or TLR9 inhibitors, enhancing mitochondrial biogenesis or mtDNA degradation, and reducing mtDNA release, may be promising therapies for these kidney diseases.

## Figures and Tables

**Table 1:** The alterations of cell-free circulating and urinary mtDNA and their correlations with acute kidney injury and chronic kidney diseases.

Disease category	Biomarker	Alteration	Disease correlation	Refs
AKI	Circulating mtDNA	Not significantly changed	Not significantly correlated with inflammation and renal injury	[16, 75, 77]
	Urinary mtDNA	Increased	Correlated positively with renal injury, negatively with eGFR and intrarenal mtDNA level	[74–77]
Nondiabetic CKD	Urinary mtDNA	Increased	Correlated positively with renal injury and eGFR decline	[88–90]
DKD	Circulating mtDNA	Decreased	Correlated negatively with inflammation and renal injury	[111]
	Urinary mtDNA	Increased	Correlated positively with inflammation and interstitial fibrosis, negatively with renal function	[108, 111]

Note: AKI, acute kidney injury; CKD, chronic kidney disease; DKD, diabetic kidney disease; eGFR, estimated glomerular filtration rate.

**Table 2:** Characteristics and implications of mtDNA-sensing pathways targeted by pharmacological modulators in acute and chronic kidney diseases.

Agent	Mechanism	Model	Administration	Effect	Refs
MitoTEMPO	Mitochondria-targeted antioxidant	Iohexol (HK-2 cell)	Preincubation for 4 h before culture with iohexol	Prevented RTEC apoptosis	[83]
		IRI (mouse)	One direct injection into each kidney after reperfusion followed by five daily i.p. injections	Ameliorated AKI	[118]
MitoQ	Mitochondria-targeted antioxidant	LPS (rat)	i.v. injection after LPS injection	Ameliorated AKI	[115]
		IRI (mouse)	i.v. injection 15 min before ischemia	Ameliorated AKI	[116]
SS-31	Mitochondria-targeted antioxidant	IRI (rat)	Subcutaneously injection 30 min before ischemia and at the onset of reperfusion	Ameliorated AKI	[117]
SkQR1	Mitochondria-targeted antioxidant	LPS (newborn rat)	i.p. administration 3 h before LPS treatment	Ameliorated AKI	[114]
Diazoxide	K_ATP_ channel opener, reducing ROS accumulation	IRI (rat)	Muscle injection before ischemia	Ameliorated AKI	[119]
Formoterol	*β*2-Adrenergic agonist, inducing MB	IRI (mouse)	i.p. injection starting at 24 h after reperfusion, daily for five days	Promoted AKI recovery	[122]
LY344864	5-HT_1F_ receptor agonist, inducing MB	IRI (mouse)	i.p. injection starting at 24 h after reperfusion, daily for five days	Promoted AKI recovery	[123]
DNase	Enzyme for mtDNA degradation	MTD (mouse)	Incubation with MTD	Abolished renal mitochondrial injury	[14]
Cyclosporin A	mPTP inhibitor	Albumin (mPTC)	Preincubation for 30 min before albumin treatment	Attenuated renal tubular injury in CKD	[95]
H154	TLR9 inhibitor	CLP (mouse)	i.p. administration immediately after CLP surgery	Ameliorated AKI	[78]
C-176	STING inhibitor	Cisplatin (mouse)	i.p. injection 1 h before cisplatin injection	Ameliorated AKI	[15]

Note: 5-HT_1F_, 5-hydroxytryptamine 1F; AKI, acute kidney injury; CKD, chronic kidney disease; CLP, cecal ligation and puncture; i.p., intraperitoneal; i.v., intravenous; IRI, ischemia/reperfusion injury; MB, mitochondrial biogenesis; mPTC, mouse proximal tubular cells; mPTP, mitochondrial permeability transition pore; MTD, mitochondrial debris; mtDNA, mitochondrial DNA; RTEC, renal tubular epithelial cells; STING, stimulator of interferon; TLR9, Toll-like receptor 9.
